# Prevalence of fecal viruses and bacteriophage in Canadian farmed mink (*Neovison vison*)

**DOI:** 10.1002/mbo3.622

**Published:** 2018-04-10

**Authors:** Xiao‐Ting Xie, Andrew M. Kropinski, Brian Tapscott, J. Scott Weese, Patricia V. Turner

**Affiliations:** ^1^ Department of Pathobiology University of Guelph Guelph ON Canada; ^2^ Ontario Ministry of Agriculture, Food and Rural Affairs (OMAFRA) Elora ON Canada

**Keywords:** antimicrobial resistance, bacteriophage, fecal virome, mink

## Abstract

Recent viral metagenomic studies have demonstrated the diversity of eukaryotic viruses and bacteriophage shed in the feces of domestic species. Although enteric disease is a major concern in the commercial mink farming industry, few etiologic agents have been well characterized. This study aimed to identify viruses shed in the fecal matter of clinically healthy commercial mink from 40 southern Ontario farms. Viral RNA was extracted from 67 pooled fecal samples (30 adult female mink and 37 kit) and amplified for Illumina sequencing on the NextSeq platform, and the resulting contigs were trimmed and assembled using Trimmomatic 0.36.0 and Spades 3.8.0 in iVirus (CyVerse, AZ, USA) and SeqMan NGen 12 (DNAStar, WI, USA). Identification of assembled sequences >100 bp (Geneious 10.1.3) showed an abundance of bacteriophage sequences, mainly from families *Siphoviridae* (53%), *Podoviridae* (22%), *Myoviridae* (20%), *Inoviridae* (1%), *Leviviridae* (0.04%), *Tectiviridae* (0.01%), and *Microviridae* (0.01%). A diverse range of vertebrate viruses were detected, of which posavirus 3, mink bocavirus, gyroviruses, and avian‐associated viruses were most abundant. Additionally, sequences from nonvertebrate viruses with water and soil‐associated amebal and algal hosts were also highly prevalent. The results of this study show that viruses shed in the fecal matter of healthy commercial mink are highly diverse and could be closely associated with diet, and that more research is necessary to determine how the detected viruses may impact mink health.

## 1 INTRODUCTION

In 2015, the Canadian (CAD) commercial mink industry had a value of $98 million in pelt sales, with approximately three million mink pelts produced (Statistics Canada, 2016b, Table 003‐0015; Statistics Canada, 2016a, Table 003‐0014). Of the 213 CAD mink farms, 46 farms are located in Ontario (Statistics Canada, 2016b, Table 003‐0015). Enteritis in mink is a generalized condition thought to be caused by multiple viral and bacterial agents, and outbreaks of diarrhea on farms pose significant economic risk to producers (Englund, Chriel, Dietz, & Hedlund, 2002; Gorham et al., 1990). Viral agents known to cause enteric disease in mink include astrovirus, rotavirus, and mink enteritis virus (Arnold, Collier, Balows, & Sussman, 1997; Englund et al., 2002; Otto et al., 2015; Reynolds, 1969; Wang et al., 2015) although the prevalence of these viruses has not been monitored in Canada.

Recent viral metagenomic (virome) studies have revealed that domestic and wild animals harbor a wide variety of divergent and novel viral species and strains, as well as viruses previously characterized and associated with disease (Bodewes et al., 2014; Duarte et al., 2013; Fehér et al., 2014; Martella et al., 2011; Ng et al., 2014; Shan et al., 2011; Zhang et al., 2014). These studies have highlighted the similarity of viromes between species with comparable diets (carnivores, omnivores), and the high prevalence of zoonotic viruses, such as hepatitis E virus (rabbits, swine) and human gyroviruses (ferret) (Fehér et al., 2014; Kasorndorkbua et al., 2004; Lhomme et al., 2013). In addition to mammalian viruses, many viral metagenomic studies have also reported a high prevalence of insect‐associated viruses and bacteriophages (Colomer‐Lluch, Jofre, & Muniesa, 2011; Fancello et al., 2014; Rolain, Fancello, Desnues, & Raoult, 2011).

As mink mortality is a production concern, identifying viruses that may play a role in mink health and disease would further the understanding of agents involved in mink enteritis and lead to the development of improved monitoring and treatment strategies. Additionally, assessment of prevalent bacteriophages may provide insight into the bacterial populations that can cause disease in mink, and help to understand the relationship between phage and bacterial populations. The objective of this study is to identify the prevalent mammalian, environmental, and phage viruses shed in the feces from clinically healthy commercial adult female mink and mink kits from 40 Ontario farms.

## 2 MATERIALS AND METHODS

### 2.1 Sample collection, dilution, and filtration

Sixty‐seven pooled fecal samples were collected between July and October of 2014 from 40 Ontario mink farms. Thirty‐seven pooled kit fecal samples and 30 pooled adult female fecal samples were collected from under three pens, representing up to three adult female mink per sample or up to 15 mink kits per sample. Information on farm location, recent history of antimicrobial use, and mink coat color was collected for each farm. Samples were collected in plastic bags and stored at −80°C until processed. To prepare a 10% fecal sample dilution, the samples were thawed and mixed thoroughly in the bag, and then 1 g of fecal matter was added to 9 ml of phosphate‐buffered saline. The sample was then centrifuged at 10,000× *g* for 15 min at 4°C to remove large particulates and bacteria. The supernatant was removed, filtered (Millipore syringe 0.45 μm filters), and stored at −20°C.

### 2.2 Purification and extraction of viral nucleic acids

To reduce nonviral nucleic acids, 200 μl of filtered supernatant was treated with a nuclease mixture of 7 μl TURBO DNase (Ambion, Life Technologies, Grand Island, NY, USA), 3 μl Baseline‐ZERO DNase (Epicentre, Chicago, IL, USA), and 1 μl of diluted RNase T1 (Fermentas Canada Inc., Burlington, ON) in 7 μl 1× DNase buffer (Ambion). This mixture was incubated at 37°C for 90 min (Victoria et al., 2009; Zhang et al., 2014). DNase and Baseline‐Zero were inactivated by incubating for 20 min at 70°C. RNase T1 was inactivated during the first step of nucleic acid extraction. Viral nucleic acids were extracted from 200 μl of the DNase‐ and RNase‐treated product (Invitrogen Viral RNA/DNA Extraction kit; ThermoFisher Scientific, Mississauga, ON, Canada). In the purification procedure, 20 μl of RNase‐free water was used to elute nucleic acids.

### 2.3 Viral cDNA synthesis and preamplification enrichment of viral cDNA and DNA

Ten microliter of extracted viral nucleic acids was incubated with 100 pmol of a primer consisting of a fixed 18 bp sequence with a random nonamer at the 3′ end (GCCGACTAATGCGTAGTCNNNNNNNNN) for 2 min at 85°C. cDNA synthesis was performed using reverse transcriptase from the QuantiTect Reverse Transcription kit (Qiagen, Mississauga, ON, Canada) according to manufacturer's instructions. For pre‐PCR amplification enrichment of viral cDNA and DNA, 10 μl of the cDNA synthesis product was incubated with 50 pmol of the previously described random primer at 92°C for 2 min, 4°C for 2 min, then with 5 U of Klenow fragment with 1× Klenow Buffer (New England Biolabs, Ipswich, MA, USA) at 37°C for 1 h (Li et al., 2015). A subset of randomly selected samples (16/67) were used to test for bacterial contamination using 16S real‐time PCR using methods described by Kobayashi et al. (2006).

### 2.4 PCR amplification and product purification

Klenow products were PCR amplified using KAPA 2G HotStart ReadyMix (Kapa Biosystems, Boston, MA, USA). Five microliter of the Klenow product was mixed with 1 μl of 2.5 mM a primer containing only the 18 bp fixed portion (GCCGACTAATGCGTAGTC) of the previously described primer. An additional 1 μl of 25 mM of MgCl_2_ was added to the KAPA master mix. Temperature cycling was performed as follows: 1 cycle of 95°C for 5 min, 33 cycles of 95°C for 30 s, 55°C for 30 s, and 72°C for 90 s. Samples were kept at 72°C for an additional 10 min of extension and held at 4°C at the end of the run. PCR products were purified once using the Agencourt AMPure XP beads (Beckman Coulter, Brea, CA, USA) with a 0.8:1 ratio of beads to sample. Eighty percent ethanol was used for the ethanol wash and 32 μl of elution buffer was used to extract purified DNA fragments from the beads.

### 2.5 NGS library preparation and sequence data analyses

Sixty‐seven samples (weaned kit *n* = 37, adult female *n* = 30) were prepared for NGS (next generation sequencing) using Nextera XT DNA Sample Preparation Kit (Illumina, San Diego, CA, USA). Samples were sequenced using Illumina NextSeq500 V2 chemistry on a 2 × 125 cycle (Donnelly Centre, Toronto, ON, Canada), and reads were demultiplexed by Donnelly software. Low quality reads were filtered using Trimmomatic 0.36.0 in iVirus (CyVerse, AZ, USA) using default parameters. Trimmomatic output was used for de novo assembly in Spades 3.8.0 (CyVerse) using kmer size 65, and SeqMan NGen 12 (DNAStar, Madison, WI, USA) (Zhang et al., 2014). Assembled contigs >700 bp were aligned to the NCBI viral reference database (viral1.1.genomic.fna.gz) using BLASTn in Geneious 10.1.3 (Biomatters Ltd, Auckland, New Zealand) with an *E* value cut‐off 10^−4^. The resulting reads that aligned over at least 100 bp with a reference viral sequence were compiled and used for further analysis. Top phage and nonphage viral families were identified for all sample libraries, and the sequences from specific viruses which had the highest prevalence were compared between for adult females and kits, and between farms grouped based on five geographical regions using JMP Software (SAS Institute, Cary, NC, USA) (Figure S1). The most prevalent vertebrate virus sequences were further assessed based on identity, sequence length, and prevalence across samples. Viral sequences with lower levels of similarity in amino acid identity (average identity <90%) were then compared to these reference viral sequences (GenBank) to identify the level of identity of protein‐encoding genes. All detected sequences for each virus with low average identity were used for de novo assembly in Geneious 10.1.3, followed by phylogenetic analysis in phylogeny.fr with their closest related viral sequences (BLASTn hits with the highest identity) (Dereeper et al., 2008).

### 2.6 Antimicrobial testing of bacterial isolates

A total of 154 pooled fecal samples collected in 2016 (*n* = 76) and 2017 (*n* = 78) were used for antimicrobial resistance (AMR) testing of *Escherichia coli* and *Salmonella* isolates. The pooled fecal sample was thoroughly mixed in the collection bag, then 1 g was aliquoted into a sterile bag for AMR testing at Public Health Agency of Canada (PHAC, National Microbiology Laboratory, Guelph, ON, Canada) using the culturing and testing methods described in the Canadian Integrated Program for Antimicrobial Resistance Surveillance (Government of Canada, 2016). Outcomes of AMR testing were evaluated using upper (UL) and lower (LL) Sterne limits. Only isolates classified as intermediate resistance (I) or resistant (R) were used for further comparisons.

### 2.7 Statistical analysis

JMP (SAS Institute) was used to conduct one‐way nonparametric Wilcoxon tests to compare the relative abundances of top phage and mammalian viral sequences between adult female mink and mink kits. For all statistical tests conducted, a *p*‐value ≤ .05 is considered significant. Information collected on mink coat color was not used for statistical analysis due to inconsistent sampling.

## 3 RESULTS

### 3.1 Prevalent phage sequences

A total of 308,817,457 sequences (average 4,609,216/sample) were used for trimming and de novo assembly. After assembly, 345,127 contigs (>700 bp) were extracted for comparison to GenBank's viral database. Contigs with ≥100 bp (112,144) of detectable similarity to a reference genome were used for further analysis. Ninety‐eight percent of reads ≥100 bp aligned to bacteriophage sequences (109,612 sequences). Phage sequences were compiled and analyzed based on bacterial host. The most prevalent sequences were identified to have *Bacillus*,* Clostridium*,* Enterococcus*,* Escherichia*,* Lactobacillus*,* Lactococcus*,* Proteus*,* Pseudomonas*,* Salmonella*,* Shigella*,* Staphylococcus*, and *Streptococcus* bacterial hosts, with the top three bacteriophage species from each group listed in Table 1. *Escherichia* and *Enterococcus*‐associated bacteriophage sequences had the highest identities to GenBank reference sequences (84%–94%).

**Table 1:** Twelve most prevalent bacteriophage sequences in mink fecal samples based on bacterial host

Phage group	% of total phage sequences (109,612)	Species detected	Top 3 most prevalent species	Average identity (%)	Accession	Family
*Escherichia* phage	16	228	*Enterobacteria* phage phiEcoM‐GJ1	85	EF460875	*Myoviridae*
			*Enterobacteria* phage RTP	84	AM156909	*Siphoviridae*
			*Enterobacteria* phage vB_EcoS_NBD2	72	KX130668	*Siphoviridae*
*Enterococcus* phage	11	44	*Enterococcus* phage EFDG1	87	KP339049	*Myoviridae*
			*Enterococcus* phage IME_EF3	84	KF728385.2	*Siphoviridae*
			*Enterococcus* phage VD13	86	KJ094032.2	*Siphoviridae*
*Bacillus* phage	7	94	*Bacillus* phage B103	69	X99260	*Podoviridae*
			*Bacillus* phage BCJA1c	71	AY616446	*Siphoviridae*
			*Bacillus* phage vB_BhaS‐171	69	KU160496	*Siphoviridae*
*Staphylococcus* phage	6	121	*Staphylococcus* phage 6ec	72	KJ804259	*Siphoviridae*
			*Staphylococcus* phage vB_SepS_SEP9	72	KF929199	*Siphoviridae*
			*Staphylococcus* phage Stau2	71	KP881332	*Myoviridae*
*Lactococcus* phage	6	83	*Lactococcus* phage Tuc2009	71	AF109874.2	*Siphoviridae*
			*Lactococcus* phage 1706	69	EU081845	*Siphoviridae*
			*Lactococcus* phage GE1	72	KT339177	*Siphoviridae*
*Pseudomonas* phage	4	105	*Pseudomonas* phage Pf3	70	M11912	*Inoviridae*
			*Pseudomonas* phage vB_PsyM_KIL1	73	KU130126	*Myoviridae*
			*Pseudomonas* phage JBD44	71	KU199710	*Siphoviridae*
*Streptococcus* phage	4	86	*Streptococcus* phage phiARI0923	70	KT337370	*Siphoviridae*
			*Streptococcus* phage SpSL1	72	KM882824	*Siphoviridae*
			*Streptococcus* virus 9872	71	KU678390	*Siphoviridae*
*Salmonella* phage	4	80	*Salmonella* phage 9NA	69	KJ802832	*Podoviridae*
			*Salmonella* phage 64795_sal3	86	KX017520	*Siphoviridae*
			*Salmonella* phage IME207	84	KX523699.2	*Siphoviridae*
*Clostridium* phage	3	51	*Clostridium* phage 39‐O	89	EU588980	*Siphoviridae*
			*Clostridium* phage c‐st	74	AP008983	*Myoviridae*
			*Clostridium* phage phiCT19406C	72	KM983332	*Siphoviridae*
*Lactobacillus* phage	3	45	*Lactobacillus* phage phiJL‐1	72	AY236756	*Siphoviridae*
			*Lactobacillus* phage AQ113	69	HE956704	*Myoviridae*
			*Lactobacillus* phage PLE3	76	KU848186	*Siphoviridae*
*Proteus* phage	2	9	*Proteus* phage PM 75	88	KM819694	*Podoviridae*
			*Proteus* phage PM16	64	KF319020	*Podoviridae*
			*Proteus* phage vB_PmiM_Pm5461	75	KP890823	*Myoviridae*
*Shigella* phage	2	21	*Shigella* phage pSf‐1	84	KC710998	*Siphoviridae*
			*Shigella* phage pSf‐2	88	KP085586	*Siphoviridae*
			*Shigella* phage SP18	94	GQ981382	*Myoviridae*

The top three most prevalent phage species from each phage group and their respective life cycles are listed.

Seven viral families were identified in the top 12 most prevalent bacteriophage groups (76,558 sequences), including *Siphoviridae* (53%), *Podoviridae* (22%), *Myoviridae* (20%), *Inoviridae* (1%), *Leviviridae* (0.04%), *Tectiviridae* (0.01%), and *Microviridae* (0.01%). An additional 4.8% of detected bacteriophage sequences were unclassified, with the majority belonging to the order *Caudovirales*. *Pseudomonas* phage sequences were found to be significantly higher in adult female mink samples (*p* = .02), but no other significant differences were found in other detected phage sequences between age groups.

### 3.2 AMR and bacterial contamination testing

*E. coli* was successfully isolated from 22 samples of the 154 pooled 2016 and 2017 fecal samples for AMR testing (12 from 2016 and 10 from 2017), no *Salmonella* isolates were obtained. Seven *E. coli* isolates were found to have intermediate resistance or were resistant to at least one of the tested antimicrobials (three from 2016 and four from 2014), with six of seven isolates found to be resistant to tetracycline (Table 2). The remaining 15 isolates were not found to be resistant to any of the tested antimicrobials. The samples (16/67 sequenced samples) randomly selected for 16S rt‐PCR were negative for bacterial contamination.

**Table 2:** Antimicrobial resistance testing of *Escherichia coli* isolates from pooled adult female mink fecal samples collected in 2016 and 2017, where dashes represent susceptible isolates, I represents isolates with intermediate resistance, and R represents resistant isolates

	Isolate ID						
	160008	160028	160055	170015	170055	170056	170059
Amoxicillin/clavulanic acid	—	—	—	—	—	>32, R	—
Ampicillin	—	—	—	—	>32, R	>32, R	—
Cefoxitin	—	—	—	—	—	>32, R	—
Ceftriaxone	—	—	—	—	—	2, I	—
Ciprofloxacin	—	0.12, I	—	—	—	—	—
Gentamicin	—	—	—	—	>16, R	>16, R	—
Streptomycin	—	64, R	—	—	64, R	64, R	—
Sulfisoxazole	>256, R	—	—	—		>256, R	>256, R
Tetracycline	>32, R	—	>32, R	>32, R	>32, R	>32, R	>32, R
Trimethoprim/sulfamethoxazole	>4, R	—	—	—	—	—	>4, R

### 3.3 Prevalent nonphage viral sequences

Of 2,532 nonphage sequences, 49% (1,237) aligned to vertebrate viruses. The most prevalent of the vertebrate viruses were from viral families *Parvoviridae*,* Circoviridae*,* Genomoviridae*, and *Herpesviridae*. Vertebrate viral sequences detected with the highest identity (>92%) to previously reported viruses include posavirus 3, mink bocavirus, chicken anemia virus, avian gyrovirus 2, avian adeno‐associated virus strains DA‐1 and ATCC VR‐865, gyrovirus 4, gyrovirus GyV3 (Table 3). Sequences with relatively low identity to saimiriine herpesvirus 2, chimpanzee feces‐associated virus 1, *Gemykibivirus* HCBI8.215 virus, *Desmodus rotundus* parvovirus, gyrovirus Tu243, chicken parvovirus ABU‐P1 and chicken‐associated smacovirus were also detected in high numbers (Table 4). No significant differences were found when considering vertebrate viral sequence prevalence between female mink and kit samples or between farm groups.

**Table 3:** Detected vertebrate viruses with the highest identity to previously reported viruses and their prevalence in samples

Detected virus	Accession number	% of total vertebrate viral sequences (1,237)	Average identity % (range)	Prevalence in samples (%, *n* = 67)
Posavirus 3 strain 958‐4	KR019688	11	93 (84–96)	7
Mink bocavirus	KU950356	11	98 (74–100)	49
Chicken anemia virus	NC001427	7	97 (73–99)	63
Avian gyrovirus 2	HM590588	4	97 (91–100)	54
Avian adeno‐associated virus strain DA‐1	AY629583	3	92 (70–98)	43
Avian adeno‐associated virus ATCC VR‐865	AY186198	2	92 (77–97)	28
Gyrovirus 4 strain RS/BR/15	KY024580	0.3	96 (89–100)	6
Gyrovirus GyV3	JQ308210	0.3	94 (81–99)	6

**Table 4:** Detected vertebrate viral sequences with low identity to previously reported viruses, their prevalence in 67 pooled mink fecal samples, and the protein‐encoding genes detected in the query sequences

Detected virus	Accession number	% of total vertebrate viral sequences (1,237)	Prevalence in samples (%)	Average identity (%)	Sequence encoded proteins (% identity)
Saimiriine herpesvirus 2	AH003100.2	7	52	71	Thymidylate synthase (100%)
Chimpanzee feces‐associated virus 1 CPNG_29286	KR704911	5	43	70	Replication‐associated proteins (100%)
HCBI8.215 virus	LK931483	4	25	89	Capsid and replication‐associated proteins (100%)
*Desmodus rotundus* parvovirus strain DRA25	KX907333	3	39	68	NS1 and capsid protein 1 (100%)
Gyrovius Tu243	KF294861	3	39	67	VP1 and VP2 (100%)
Chicken parvovirus ABU‐P1	GU214704	2	24	71	NS1, VP1 and VP2 (100%)
Chicken‐associated smacovirus strain RS/BR/2015/4	KY086299	2	22	89	Capsid and replication‐associated proteins (100%)

In addition to vertebrate viruses, 51% (1,295/2,532) of nonphage sequences were associated with nonvertebrate hosts, including environment‐associated viruses (water, algae and soil), and plant, insect, fungal, or crustacean‐associated hosts. The 10 most prevalent of the nonvertebrate viral sequences include algae, ameba, insect, and crustacean‐associated viruses, with 18%–63% prevalence in samples (Table 5). Megavirus courdo11 and Tokyovirus A1 are water‐associated amebal viruses, whereas mimivirus terra2 is a soil‐associated ameobal virus. *Cafeteria roenbergensis* virus BV‐PW1, *Aureococcus anophagefferens* virus isolate BtV‐01, *Chrysochromulina ericina* virus isolate CeV‐01B, and *Acanthocystis turfacea Chlorella* virus 1 are water‐associated viruses with algae as hosts. White spot syndrome virus strain CN01 affects shrimp, whereas *Culex pipiens* densovirus and *Melanoplus sanguinipes* entomopoxvirus are associated with mosquitos and grasshoppers, respectively.

**Table 5:** Top 10 most prevalent non‐vertebrate viral sequences detected in 67 pooled mink fecal samples

Detected virus	Accession number	% of total non‐phage sequences (2,532)	Average identity (%)	Prevalence in samples (%)
Mimivirus terra2	KF527228	10	79	63
Megavirus courdo11	JX975216	8	82	58
*Cafeteria roenbergensis* virus BV‐PW1	GU244497	7	82	24
White spot syndrome virus strain CN01	KT995472	5	73	19
*Aureococcus anophagefferens* virus isolate BtV‐01	KJ645900	5	76	46
*Chrysochromulina ericina* virus isolate CeV‐01B	KT820662	5	74	61
Tokyovirus A1	AP017398	2	79	40
*Culex pipiens* densovirus	FJ810126	1	69	30
*Acanthocystis turfacea* Chlorella virus 1	AY971002	1	71	18
*Melanoplus sanguinipes* entomopoxvirus	AF063866	1	82	18

### 3.4 Analysis of vertebrate viral sequences with low average identity

**Figure 1:**
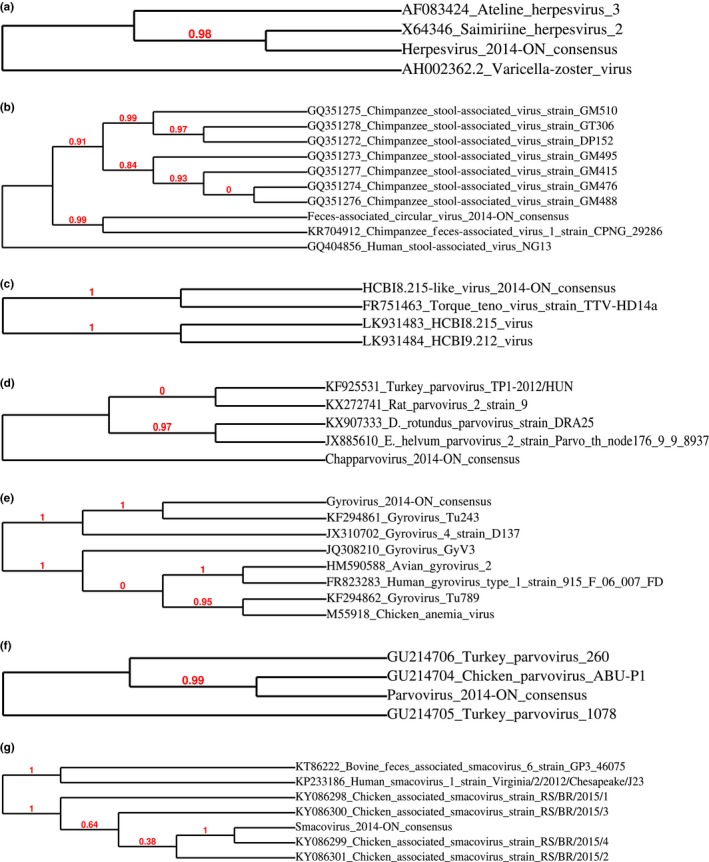
Phylogenetic comparison of consensus sequences to related viruses (a) Herpesvirus 2014‐ON consensus (b) Feces‐associated circular virus 2‐14‐ON consensus (c) HCBI8.215‐like virus 2014‐ON consensus (d) Chapparvovirus 2014‐ON consensus (e) Gyrovirus 2014‐ON consensus (f) Parvovirus 2014‐ON consensus (g) Smacovirus 2014‐ON consensus

This study identified sequences from seven prevalent viruses that had low average identity (<90%) to the reference sequences of vertebrate viruses. The average identities of detected sequences, their prevalence in samples, as well as query‐encoded proteins are listed in Table 4. Figure 1a shows the phylogenetic relationship between the consensus sequence (Herpesvirus 2014‐ON_consensus) from de novo assembly of all detected sequences with similarity to saimiriine herpesvirus 2 and viruses with similar genome structure. Herpesvirus 2014‐ON_consensus was most closely related saimiriine herpesvirus 2 and also clustered closely with ateline herpesvirus 3 (AF083424), but was distinct from varicella‐zoster virus (AH002362.2) (Albrecht et al., 1992). Analysis of individual viral sequences with highest similarity to an unclassified virus, chimpanzee feces‐associated virus 1 CPNG_29286 (KR704911), showed segments with 100% and 76% identity to genes encoding replication‐associated proteins in chimpanzee feces‐associated circular DNA virus 1 CPNG_29286 and chimpanzee feces‐associated circular DNA virus 1 CPNG_29268 (KR704711). Figure 1b shows that the assembled consensus sequence (feces‐associated circular virus 2‐14‐ON_consensus) clusters closely with 8 other strains of chimpanzee stool‐associated circular viruses (GQ351272–GQ351278, KR704912), but was distinct from human stool‐associated circular virus NG13 (GQ404856). Figure 1c shows that the assembled consensus sequence (HCBI8.215‐like virus 2014‐ON_consensus) are more closely related to torque teno virus strain TTV‐HD14a (FR751463) compared to HCBI8.215 virus (LK931483) and HCBI9.212 virus (LK931484). Sequences with highest similarity to *Desmodus rotundus* parvovirus strain DRA25 (KX907333, *Parvoviridae*) were used in de novo assembly, resulting in chapparvovirus 2014‐ON_consensus. This consensus sequence was not closely related to the *Desmodus rotundus* parvovirus strain DRA25 genome or three other parvoviruses with similar structure (KF925531, KX272741, and JX885610) (Figure 1d) (De Souza et al., 2017). Sequences with highest similarity to gyrovirus Tu243 (KF294861, *Circoviridae*) had segments with 100% identity to gyrovirus Tu243 VP1 and VP2 genes, as well as 63% identity to the VP1 gene of gyrovirus 4. The longest sequence had 64% identity over 76% (1,530/2,020) of the gyrovirus Tu243 genome. Phylogenetic analysis shows that assembled sequence (gyrovirus 2014‐ON_consensus) clustered most closely with gyrovirus Tu243 and gyrovirus 4 strain D137 (JX310702), and was also closely related to gyrovirus GyV3 (JQ308210), avian gyrovirus 2 (HM590588), human gyrovirus 1 strain 915 F 06 007 FD (FR823283), gyrovirus Tu789 (KF294862), and chicken anemia virus (M55918) (Figure 1e) (Chu et al., 2013; Phan et al., 2014; Sauvage et al., 2011). Segments of detected sequences had 100% identity to NS1, VP1, and VP2 proteins. The assembled sequences with highest similarity to chicken parvovirus ABU‐P1 (GU214704, *Parvoviridae*) (parvovirus 2014‐ON_consensus) was highly related to the genomes of chicken parvovirus ABU‐P1 and turkey parvovirus 260 (GU214706), which clustered separately from turkey parvovirus 1,078 (GU214705) (Figure 1f) (Day & Zsak, 2010). Sequences with highest similarity to chicken‐associated smacovirus strain RS/BR/2015/4 (KY086299, unclassified) encoded for capsid and replication‐associated proteins with 100% and 95% identities to chicken‐associated smacovirus strain RS/BR/2015/4 and human smacovirus (AJF23075). In phylogenetic analysis, smacovirus 2014‐ON_consensus sequence clustered most closely with chicken‐associated smacovirus strains RS/BR/2015/1–RS/BR/2015/4 (KY086298–KY086301), but were also closely related to bovine feces‐associated smacovirus strain GP3_46075_cow (KT86222) and human smacovirus 1 isolate Virginia/2/2012/Chesapeake/J23 (KP233186) (Figure 1g).

## 4 DISCUSSION

This study is a preliminary assessment of viral sequences in the fecal matter of healthy commercial mink on 40 Ontario farms, and the diversity of bacteriophage and eukaryotic virus sequences was fairly consistent with previous research on the fecal virome of carnivorous species (Fehér et al., 2014; Ng et al., 2014; Phan et al., 2015; Smits et al., 2013; Zhang et al., 2014). The 12 most prevalent phages detected in this study represent 70% (76,558/109,612) of all detected phage sequences. Comparison between the phage sequences and their respective bacterial hosts in the same cohort of mink fecal samples show that *Enterococcus*,* Lactobacillus*,* Lactococcus*,* Clostridium*,* Escherichia*,* Streptococcus,* and *Pseudomonas* species were also prevalent in the mink microbiome (unpublished data). *Bacillus*,* Salmonella*,* Shigella*,* Staphylococcus*, and *Proteus* bacterial populations were not found to be highly prevalent in the mink fecal microbiome study (unpublished data). In a fecal microbiome study conducted on samples from mink in Northeast China, Zhao et al. (2017) showed that the two most prevalent bacterial genera were *Clostridium* and *Escherichia*, phages, both of which were found in our study. Interestingly, significantly higher numbers of *Pseudomonas*‐associated phage sequences were detected in adult female fecal samples compared to kit samples (*q* = 0.02), but since the detected sequences may not represent colonization by *Pseudomonas* species, the implications of these results remain unclear. Although previous studies have shown that lytic phage therapies may be useful in controlling *Pseudomonas* bacterial populations (Cao et al., 2015; Gu et al., 2016), further investigation is required to understand the natural role that the associated bacteriophage species play in bacterial populations. Producers were asked to voluntarily report the use of antimicrobials on farms, but due to only partial completion of the survey, the information collected on antimicrobial use from the 2014 sample cohort may not be fully representative. Therefore, any relationship between antimicrobial use and the relative abundance of targeted bacterial species could not be analyzed.

This study found the highest number of vertebrate viral sequences from the families *Herpesviridae*,* Parvoviridae*,* Circoviridae*,* Anelloviridae,* and *Picornaviridae*. Previous fecal virome studies in ferrets and felids have also found high numbers of viral sequences belonging to the families *Parvoviridae*,* Anelloviridae,* and *Picornaviridae*, but have also detected sequences from the families *Astroviridae*,* Reoviridae*,* Hepeviridae*,* Papillomaviridae*,* Picobirnaviridae*, and *Coronaviridae* (Fehér et al., 2014; Ng et al., 2014; Smits et al., 2013; Zhang et al., 2014). High numbers of sequences with 84%–96% identity to posavirus 3 strain 958‐4 were identified, which has been previously detected in fecal samples collected from commercial swine in high animal density farms (Hause, Hesse, & Anderson, 2015). Hause et al. (2015) suggest that this strain of posavirus is derived from nematodes parasitizing commercial swine. The detected posavirus sequences may be the result of contamination from the soil at the time of fecal sample collection, but also could be attributed to the mink diet, which often consists of pork and poultry products, or nematode infections in the mink gut (Krog, Breum, Jenson, & Larsen, 2013). Similarly, the identified avian‐associated viral sequences (chicken anemia virus, parvovirus, smacovirus, and avian adeno‐associated virus) were most likely the result of mink diet. Previous evidence from viral metagenomic studies and case reports in ferrets, felids, mink, and other wild carnivores have also hypothesized that the presence of avian viruses and swine viruses in fecal samples is due to the diet of the animals (Bodewes et al., 2014; Fehér et al., 2014; Krog et al., 2013; Smits et al., 2013). Further research is required to determine the correlation between diet and the fecal virome of mink. This is also the first report of mink bocavirus sequences in commercial mink fecal samples in Canada, with 98%–100% identity to the strain identified in 2016 in China (Yang et al., 2016). This strain was most closely related to feline bocavirus (JQ692585). Yang et al. (2016) found no correlation between mink bocavirus and diarrhea, but stated that these results may not be fully representative due to the small sample size.

Viruses with low average identity were used in de novo assembly and the resulting consensus sequences were compared to closely related viruses. Most consensus sequences found to be closely related to the initial best BLASTn hit of the individual sequences, with the exception of HCBI8.215‐like virus 2014‐ON_consensus and chapparvovirus 2014‐ON_consensus. HCBI8.215‐like virus 2014‐ON_consensus was found to be more closely related to torque teno virus strain TTV‐HD14a. Chapparvovirus 2014‐ON_consensus did not cluster with the initial best BLASTn hit, *Desmodus rotundus* parvovirus strain DRA25, or three other parvoviruses with similar not closely related to *Eidolon helvum* parvovirus 2 isolate Parvo_th_node176_9_9_893755, rat parvovirus 2 strain 9 or turkey parvovirus TP1‐2012/HUN, indicating that it could be a novel mink parvovirus. Aside from mink bocavirus, the other prevalent vertebrate viruses identified in this study have been previously isolated in other species. HCBI8.215 virus was first isolated from the serum of healthy cattle, and gyrovirus Tu243 and GyV3 were isolated from human fecal samples (Lamberto, Gunst, Muller, Hausen, & de Villiers, 2014; Phan et al., 2012, 2014). Six of the 15 prevalent vertebrate viruses described in this study are of avian origin. Although virus shedding does not represent active infections, some of the viruses identified in this study may have the potential to be transmitted to the humans, commercial and wild animals in close proximity to mink farms due to poor biosecurity (Compo et al., 2017).

In conclusion, this viral metagenomic study provides a preliminary overview of the commercial mink fecal virome, showing a diverse range of bacteriophage and eukaryotic virus sequences, including a potentially novel chapparvovirus. It is not known whether the detected bacteriophage and eukaryotic virus sequences represent commensal species, or if these viruses are capable of influencing bacterial populations and causing disease in mink. Further research is required to clarify the phylogeny of low‐identity sequences identified in this study and to determine the role of these prevalent viruses in mink health.

## AUTHOR CONTRIBUTION

P.V.T., B.T., and J.S.W. conceived of the work and prepared the grant; X.T.X., B.T., A.K., and J.S.W. conducted the work and analyzed the data; X.T.X. and P.V.T. co‐wrote the paper, and all authors contributed to manuscript review.

## CONFLICT OF INTEREST

The authors declare no conflicts of interest.
