# Supplementary figures and images for: Prevalence of fecal viruses and bacteriophage in Canadian farmed mink (Neovison vison)
Source: Microbiologyopen. 2018 Apr 10;8(1):e00622. doi: 10.1002/mbo3.622 (PMC6341152; doi:10.1002/mbo3.622)

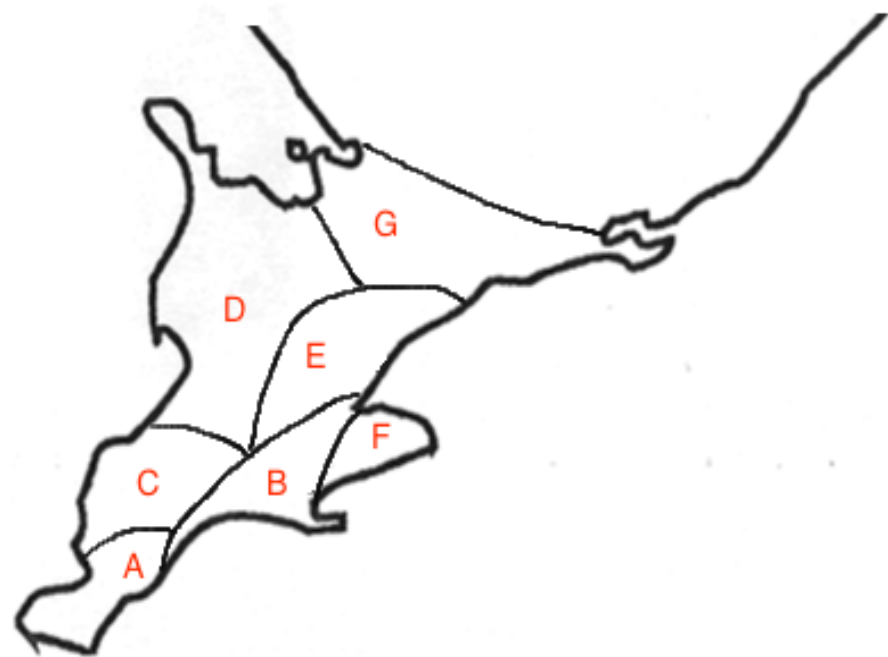

Supplement: Supplementary file 1 [file MBO3-8-e00622-s001.pdf]
