# Supplementary material for: Prevalence of fecal viruses and bacteriophage in Canadian farmed mink (Neovison vison)
Source: Microbiologyopen. 2018 Apr 10;8(1):e00622. doi: 10.1002/mbo3.622 (PMC6341152; doi:10.1002/mbo3.622)
Supplement: Supplementary file 2 [file MBO3-8-e00622-s002.docx]

**Additional Supporting Information.**

**Table S1.** All detected phage groups based on bacterial host and the top species identified (109,612).

**Table S2**. All detected vertebrate viral sequences (1,237).

**Table S3**. All detected non-vertebrate eukaryotic viral sequences, including water, soil, algae, insect, fungal, and crustacean-associated viruses (1,295).

**Figure S1.** Geographical grouping of 40 commercial mink farms in Ontario, where farms were grouped into 7 groups (A-G) based on geographical proximity to each other.

**Table S1.** All detected phage groups based on bacterial host and the top species identified (109,612).

| Phage group | Sequences detected | Top species |
| --- | --- | --- |
| *Escherichia* phage | 17542 | *Enterobacteria* phage phiEcoM-GJ1 |
| *Enterococcus* phage | 12470 | *Enterococcus* phage EFDG1 |
| *Bacillus* phage | 7260 | *Bacillus* phage B103 |
| *Staphylococcus* phage | 6510 | *Staphylococcus* phage 6ec |
| *Lactoccocus* phage | 6184 | *Lactococcus* phage Tuc2009 |
| *Streptococcus* phage | 4779 | *Streptococcus* phage phiARI0923 |
| *Pseudomonas* phage | 4753 | *Pseudomonas* phage Pf3 |
| *Salmonella* phage | 4247 | *Salmonella* phage 9NA |
| *Clostridium* phage | 3319 | *Clostridium* phage 39-O |
| *Lactobacillus* phage | 2884 | *Lactobacillus* phage phiJL-1 |
| *Proteus* phage | 2439 | *Proteus* phage PM 75 |
| *Shigella* phage | 2389 | *Shigella* phage pSf-1 |
| *Acinetobacter* phage | 2382 | *Acinetobacter* phage LZ35 |
| *Geobacillus* phage | 2356 | *Geobacillus* phage GBK2 |
| *Enterobacter* phage | 1886 | *Enterobacter* phage Tyrion |
| *Cronobacter* phage | 1766 | *Cronobacter* phage Dev-CD-23823 |
| *Pseudoalteromonas* phage | 1762 | *Pseudoalteromonas* phage H101 |
| *Arthrobacter* phage | 1741 | *Arthrobacter* phage Mudcat |
| *Listeria* phage | 1715 | *Listeria* phage A006 |
| *Cellulophaga* phage | 1711 | *Cellulophaga* phage phi10:1 |
| *Citrobacter* phage | 1448 | *Citrobacter* phage CR8 |
| *Klebsiella* phage | 1368 | *Klebsiella* phage F19 |
| *Idiomarinaceae* phage | 1302 | *Idiomarinaceae* phage 1N2-2 |
| *Persicivirga* phage | 1261 | *Persicivirga* phage P12024L |
| *Flavobacterium* phage | 1242 | *Flavobacterium* phage 11b |
| *Paenibacillus* phage | 1215 | *Paenibacillus* phage Tripp |
| *Vibrio* phage | 1208 | *Vibrio* phage N4 |
| *Erwinia* phage | 1203 | *Erwinia* phage FE44 |
| *Edwardsiella* phage | 1196 | *Edwardsiella* phage PEi21 |
| *Listonella* phage | 1193 | *Listonella* phage phiHSIC |
| *Mannheimia* phage | 1156 | *Mannheimia* phage vB_MhS_1152AP2 |
| *Rhodococcus* phage | 1128 | *Rhodococcus* phage ReqiPepy6 |
| *Mycobacterium* phage | 1111 | *Mycobacterium* phage Bane1 |
| *Croceibacter* phage | 1102 | *Croceibacter* phage P2559Y |
| *Morganella* phage | 197 | *Morganella* phage MmP1 |
| *Streptomyces* phage | 191 | *Streptomyces* phage SF3 |
| *Yersinia* phage | 174 | *Yersinia* phage Berlin |
| *Rhodobacter* phage | 167 | *Rhodobacter* phage RcRhea |
| *Kluyvera* phage | 156 | *Kluyvera* phage Kvp1 |
| Weissella phage | 146 | *Weissella* phage WCP30 |
| Pectobacterium phage | 142 | *Pectobacterium* phage PM1 |
| Parabacteroides phage | 137 | *Parabacteroides* phage YZ-2015b |
| Polaribacter phage | 128 | *Polaribacter* phage P12002L |
| Leuconostoc phage | 127 | *Leuconostoc* phage Ln-9 |
| Synechococcus phage | 127 | *Synechococcus* phage S-MbCM7 |
| *Aeromonas* phage | 124 | *Aeromonas* phage pAh6-C |
| *Brevibacillus* phage | 120 | *Brevibacillus* phage Jenst |
| *Chlamydia* phage | 117 | *Gokushovirinae* Bog1183_53 |
| *Psychrobacter* phage | 117 | *Psychrobacter* phage Psymv2 |
| *Erysipelothrix* phage | 84 | *Erysipelothrix* phage SE-1 |
| *Serratia* phage | 62 | *Serratia* phage Eta |
| *Clavibacter* phage | 40 | *Clavibacter* phage CMP1 |
| *Erwinia* phage | 13 | *Erwinia amylovora* phage Era103 |
| Iodobacteriophage | 7 | Iodobacteriophage phiPLPE |
| *Verrucomicrobia* phage | 7 | *Verrucomicrobia* phage P8625 |
| *Xanthomonas* phage | 1 | *Xanthomonas* phage vB_XveM_DIBBI |

**Table S2**. All detected vertebrate viral sequences (1,237).

| Vertebrate viral sequences | Sequences detected |
| --- | --- |
| Mink bocavirus clone 1 | 134 |
| Posavirus 3 strain 958-4 | 134 |
| Chimpanzee faeces associated circular DNA molecule 1 isolate CPNG_29268 | 119 |
| Chicken anemia virus | 84 |
| Saimiriine herpesvirus 2 complete genome | 71 |
| Avian adeno-associated virus strain DA-1 | 56 |
| Avian gyrovirus 2 | 54 |
| HCBI8.215 virus complete sequence | 48 |
| Desmodus rotundus parvovirus strain DRA25 | 46 |
| Gyrovirus Tu243 | 37 |
| Chicken parvovirus ABU-P1 | 25 |
| BeAn 58058 virus | 24 |
| Avian adeno-associated virus ATCC VR-865 | 24 |
| Chicken associated smacovirus strain RS/BR/2015/4 | 22 |
| Caribou feces-associated gemycircularvirus | 21 |
| Badger feces-associated gemycircularvirus strain 588t | 21 |
| Volepox virus strain CA | 21 |
| Ovine herpesvirus 2 strain BJ1035 | 19 |
| IAS virus | 18 |
| Ungulate bocaparvovirus 6 strain USII/03 | 18 |
| Fowl adenovirus D | 17 |
| Turkeypox virus strain TKPV-HU1124/2011 | 17 |
| Porcine stool-associated circular virus 4 isolate CP2 | 14 |
| Human herpesvirus 7 | 7 |
| Bovine faeces associated circular DNA virus 1 isolate GP3-46075_cow2 | 6 |
| Human herpesvirus 8 | 6 |
| Sheeppox virus 17077-99 | 6 |
| Deerpox virus W-848-83 | 5 |
| Gyrovirus 4 strain D137 | 5 |
| Porcine parvovirus | 5 |
| Turkey parvovirus 1078 | 5 |
| Chicken stool-associated gemycircularvirus strain RS/BR/2015 | 5 |
| Gyrovirus 4 | 4 |
| Gyrovirus GyV3 | 4 |
| Macacine herpesvirus 3 | 4 |
| Penguinpox virus isolate PSan92 | 4 |
| Rabbit fibroma virus | 4 |
| Raccoon dog amdovirus isolate HS-R | 4 |
| Bat mastadenovirus WIV12 | 4 |
| Gyrovirus Tu789 | 4 |
| Alcelaphine herpesvirus 1 | 4 |
| Cyprinid herpesvirus 3 | 4 |
| Parus major densovirus isolate PmDNV-JL | 4 |
| Alcelaphine herpesvirus 2 isolate topi-AlHV-2 | 3 |
| Duck adenovirus 2 strain GR | 3 |
| Faeces associated gemycircularvirus 21 isolate 29_Fec80018_llama | 3 |
| Human herpesvirus 6A | 3 |
| Murine roseolovirus isolate YOK1 | 3 |
| Pig stool associated circular ssDNA virus GER2011 | 3 |
| Pigeonpox virus isolate FeP2 | 3 |
| Rat stool-associated circular ssDNA virus isolate KS/11/0577 | 3 |
| Protoparvovirus HK-2014 isolate ParvoQ45/2013 | 3 |
| Badger feces-associated gemycircularvirus strain 588t | 2 |
| Bovine herpesvirus 5 | 2 |
| Bovine herpesvirus 6 isolate Pennsylvania 47 | 2 |
| Equid herpesvirus 4 | 2 |
| Felis catus gammaherpesvirus 1 isolate 31286 | 2 |
| Frog virus 3 | 2 |
| Murid herpesvirus 1 | 2 |
| Porcine circovirus 2 | 2 |
| Skunkpox virus strain WA | 2 |
| Turkey stool associated circular ssDNA virus strain TuSCV | 2 |
| Macaca nemestrina herpesvirus 7 | 2 |
| Orf virus | 2 |
| African swine fever virus strain BA71V | 1 |
| Avian paramyxovirus 2 strain APMV-2/Chicken/England/7702/06 | 1 |
| Ball python nidovirus strain 07-53 | 1 |
| Bat circovirus POA/2012/VI | 1 |
| Black howler monkey smacovirus isolate SF1 | 1 |
| Cowpox virus | 1 |
| Duck faeces associated circular DNA virus 2 isolate 4_Fec60467_duck | 1 |
| Elephant endotheliotropic herpesvirus 4 isolate North American NAP69 | 1 |
| Elephantid herpesvirus 1 | 1 |
| Equid herpesvirus 1 | 1 |
| Equid herpesvirus 8 | 1 |
| European hedgehog papillomavirus | 1 |
| Goose circovirus | 1 |
| Gyrovirus GyV7-SF | 1 |
| Human adenovirus 54 | 1 |
| Human adenovirus F | 1 |
| Human erythrovirus V9 | 1 |
| Human genital-associated circular DNA virus-1 isolate 349 | 1 |
| Human herpesvirus 3 | 1 |
| Human herpesvirus 4 | 1 |
| Human immunodeficiency virus 1 | 1 |
| Human papillomavirus type 92 | 1 |
| Human parainfluenza virus 1 | 1 |
| Human respiratory syncytial virus | 1 |
| Magpie-robin coronavirus HKU18 | 1 |
| Mouse cyclovirus isolate Cyclo-sf1 | 1 |
| Muscovy duck circovirus | 1 |
| Porcine stool-associated circular virus 6 isolate XP1 | 1 |
| Raccoonpox virus | 1 |
| Rat bocavirus strain HK1S | 1 |
| Saimiriine herpesvirus 4 strain SqSHV | 1 |
| Simian retrovirus 8 strain SRV8/SUZ/2012 | 1 |
| Squirrel poxvirus strain Red squirrel UK complete genome | 1 |
| Turkey adenovirus 1 | 1 |
| Turkey adenovirus 4 isolate TNI1 | 1 |
| Turkey adenovirus 5 isolate 1277BT | 1 |
| Adeno-associated virus - 4 | 1 |
| Adeno-associated virus 5 | 1 |
| Bovine ephemeral fever virus | 1 |
| Bovine papular stomatitis virus strain BV-TX09c1 | 1 |
| Yaba-like disease virus | 1 |
| Vaccinia virus | 1 |
| Variola virus | 1 |
| Tupaiid herpesvirus 1 | 1 |
| Sumatran orang-utan polyomavirus complete genome, isolate Pi | 1 |
| Suid herpesvirus 1 | 1 |
| Respiratory syncytial virus | 1 |
| Oropouche virus segment M | 1 |

**Table S3**. All detected non-vertebrate eukaryotic viral sequences, including water, soil, algae, insect, fungal, and crustacean-associated viruses (1,295).

| Virus | Sequences detected |
| --- | --- |
| Mimivirus terra2 | 246 |
| Megavirus courdo11 | 203 |
| Cafeteria roenbergensis virus BV-PW1 | 169 |
| Aureococcus anophagefferens virus isolate BtV-01 | 139 |
| Chrysochromulina ericina virus isolate CeV-01B | 129 |
| White spot syndrome virus strain CN01 | 116 |
| Tokyovirus A1 | 60 |
| Culex pipiens densovirus | 34 |
| Acanthocystis turfacea Chlorella virus 1 | 30 |
| Melanoplus sanguinipes entomopoxvirus | 30 |
| Choristoneura rosaceana entomopoxvirus 'L' | 22 |
| Paramecium bursaria Chlorella virus 1 | 20 |
| Cedratvirus A11 | 16 |
| Yellowstone lake mimivirus isolate: 1 | 15 |
| Solenopsis invicta densovirus isolate SiDNV-Arg | 9 |
| Amsacta moorei entomopoxvirus 'L' | 7 |
| Adoxophyes honmai enomopoxvirus 'L' | 6 |
| Choristoneura biennis entomopoxvirus 'L' | 4 |
| Hubei picorna-like virus 50 strain spider113255 | 4 |
| Anomala cuprea entomopoxvirus strain: CV6M | 3 |
| Junonia coenia densovirus | 3 |
| Lake Sarah-associated circular virus-10 isolate LSaCV-10-LSSO-2013 | 3 |
| Grapevine virus F | 3 |
| Ectocarpus siliculosus virus 1 | 2 |
| Hyposoter fugitivus ichnovirus segment C11 | 2 |
| Golden Marseillevirus | 1 |
| Grapevine fleck virus | 1 |
| Hokovirus HKV1 Hokovirus_4 | 1 |
| Hubei arthropod virus 3 strain GCM7473 | 1 |
| Hubei sobemo-like virus 24 strain WHLC5390 | 1 |
| Hubei tombus-like virus 18 strain QTM27278 | 1 |
| Hubei virga-like virus 12 strain SCM49209 | 1 |
| Mythimna loreyi densovirus | 1 |
| Narcissus symptomless virus | 1 |
| Niemeyer virus, partial genome | 1 |
| Okra yellow mosaic Mexico virus DNA B | 1 |
| Peanut chlorotic streak virus | 1 |
| Sulfolobus islandicus rudivirus 3 isolate SIRV3 | 1 |
| Sulfolobus spindle-shaped virus 7 | 1 |
| Sulfolobus turreted icosahedral virus | 1 |
| Wuhan house centipede virus 2 strain arthropodmix22554 | 1 |
| Wuhan pillworm virus 3 strain WHSFII20254 | 1 |
| Wuhan spider virus 8 strain spider134060 | 1 |
| Xestia c-nigrum granulovirus | 1 |
| Zygosaccharomyces bailii CLIB 213 | 1 |

**
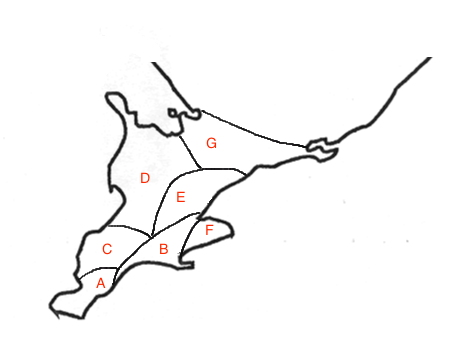
**

**Figure S1.** Geographical grouping of 40 commercial mink farms in Ontario, where farms were grouped into 7 groups (A-G) based on geographical proximity to each other.
